# Cross Regulatory Network Between Circadian Clock and Leaf Senescence Is Emerging in Higher Plants

**DOI:** 10.3389/fpls.2018.00700

**Published:** 2018-05-23

**Authors:** Yan Wang, Yuanyuan Zhang, Lei Wang

**Affiliations:** ^1^Key Laboratory of Plant Molecular Physiology, CAS Center for Excellence in Molecular Plant Sciences, Institute of Botany, Chinese Academy of Sciences, Beijing, China; ^2^University of Chinese Academy of Sciences, Beijing, China

**Keywords:** circadian clock, leaf senescence, CCA1, evening complex, MYC2

## Abstract

Circadian clock and senescence have been shown to tightly intertwined with each other in numerous eukaryotes, but the regulation of circadian oscillator on triggering leaf senescence, and vice versa, remains largely unknown in higher plants. Very recently, circadian system and leaf senescence were found to be highly interconnected in higher plants. Circadian clock was shown to regulate leaf senescence through a few cross signaling pathways including age-dependent, plant hormone mediated, and dark induced manners to trigger the onset of leaf senescence. By contrast, circadian clock itself also can be affected by the leaves senescing process. The eventually delineating cross networks between circadian clock and leaf senescence will lay the foundation for understanding the fitness of developmental dynamics of plants with aging.

## Introduction

By synchronizing the cellular events with external light-dark cycle resulted from daily rotation of earth, circadian clock regulates many aspects of plant growth and development, such as photosynthesis, pathogen defense, and flowering time, and provides a fitness benefit for higher plants (Dodd et al., 2005; Harmer, 2009; Greenham and McClung, 2015). The current consensus is that cell-autonomous core oscillator of circadian clock comprises three interlocking negative feedback loops. *CIRCADIAN CLOCK-ASSOCIATED1* (*CCA1*) and *LATE* *ELONGATED HYPOCOTYL* (*LHY*), suppress expression of *PSEUDO-RESPONSE REGULATOR7* (*PRR7*) and *PRR9*, while PRR9/7/5 can transcriptionally repress *CCA1/LHY* expression by recruiting TOPLESS family members and HISTONE DEACETLYLASE 6/19 (Wang et al., 2013), hence forming the morning loop of core oscillator (Pokhilko et al., 2012). *TOC1* (*TIMING OF CAB EXPRESSION 1*) and *CCA1*/*LHY* reciprocally represses each other to form the central loop of core oscillator. In the evening loop, *TOC1* represses the expression of *GIGANTEA* (*GI*) (Huang et al., 2012). Moreover, the evening complex (EC), consisting of EARLY FLOWERING 3 (ELF3), ELF4 and LUX ARRHYTHMO (LUX), suppresses the expression of *TOC1*, *GI*, and *PRR9* (Nusinow et al., 2011; Herrero et al., 2012).

The key components of core oscillator mediate circadian outputs by both transcriptional and post-transcriptional mechanisms via timing numerous genes expression at appropriate phase. Recently, the accumulating evidence display that many components of circadian genetic circuit also serve as harbors to directly regulate the transcription of genes involved in clock-output pathways, such as abiotic stress response, photosynthesis and flowering time (Nusinow et al., 2011; Sawa and Kay, 2011; Nakamichi et al., 2012; Liu et al., 2013, 2016; Ezer et al., 2017). Moreover, many circadian core components also have been identified as key players to regulate hypocotyls growth and flowering time through protein–protein interaction, and affecting the abundance and activity of their interacting partners (Shin et al., 2012; Soy et al., 2016; Zhu et al., 2016; Hayama et al., 2017; Martin et al., 2018). Thus, circadian core oscillator serves as a master regulator in whole life span of plant (Sanchez and Kay, 2016). However, whether and how leaf senescence is modulated by circadian clock, or vice versa, remains to be discovered.

Leaf senescence not only means the inevitably death, as double edged sword, it also can trigger the reallocation of nutrients and energy to developing tissues or storage organs, thus properly timing leaf senescence onset is critical for the seed yield, fruit ripening and biomass production, especially for annual plants (Lim et al., 2007; Wu et al., 2012). The leaf senescence is under the control of an extremely coordinated cellular nework, which integrates the environmental information and internal signals such as plant hormone signaling, nutrition and energy status. Basically, onset of the leaf senescence is managed by developmental cues, and modulated by both the outer environmental conditions and inner signals independently and interdependently, such as dark (Sakuraba et al., 2014) and multiple hormone signaling such as JAs (Qi et al., 2015; for review see Kim et al., 2015), ethylene (Li et al., 2013; Qiu et al., 2015; also see review by Iqbal et al., 2017), ABA (Zhao et al., 2016), and strigolactone (Ueda and Kusaba, 2015). Accordingly, the signaling pathways of leaf senescence can be classified to age-dependent pathway, environmental conditions induced pathway, and internal hormones induced pathway, etc. In any environmental cues or inner signals trigged leaf senescence, this degenerative process was closely regulated by the coordinated expression profile of senescence-associated genes (SAGs). The collection of SAGs in *Arabidopsis* comprised of 192 genes involved in various regulatory networks underlying leaf senescence (Liu et al., 2011; Li et al., 2014). Very recently, a few core circadian components were found to be able to regulate the messenger RNA or protein abundance of SAGs at the transcriptional and post-transcriptional levels, respectively (Shin et al., 2012; Sakuraba et al., 2014; Song et al., 2018; Zhang et al., 2018). By contrast, circadian period were dramatically altered in the different ages of leaf, indicating the tightly reciprocal regulation between circadian clock and leaf senescence exists (Kim et al., 2016). Here, we reviewed the recent progresses on the interplay between circadian clock and leaf senescence, and proposed that the regulatory network of leaf senescence and circadian system is highly interconnected in higher plants. Unraveling this underlying interconnected mechanism will contribute to understand the complex regulatory networks for fitness and adaptive advantage of higher plants.

## Circadian Clock Components Coordinates Age-Dependent Leaf Senescence

**FIGURE 1:**
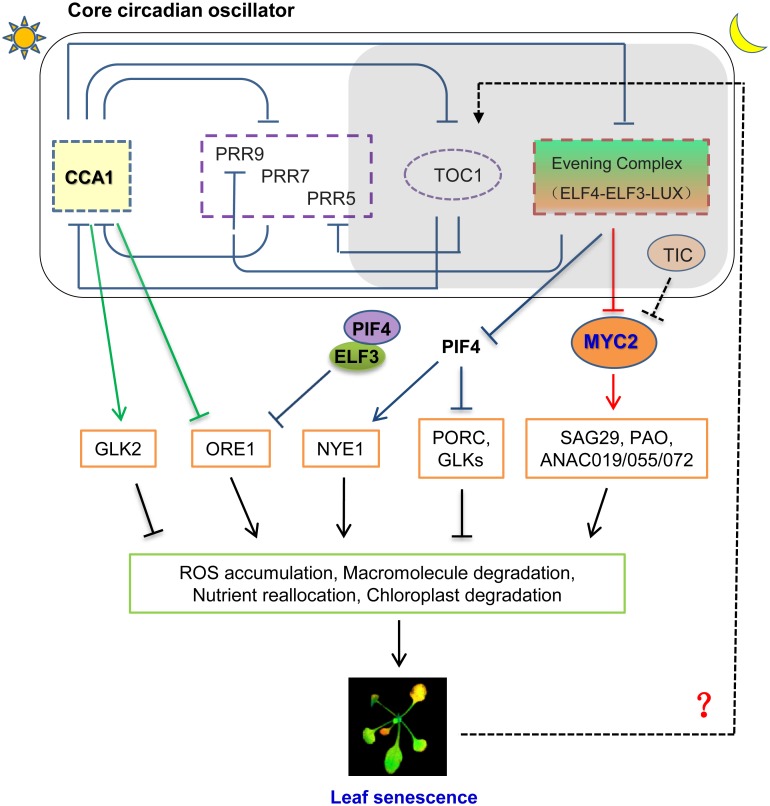
A proposed model for interplaying between circadian clock and leaf senescence in *Arabidopsis*. CCA1, one of core components of plant circadian oscillator, regulates age dependent leaf senescence by inhibiting *ORE1* and activating *GLK2* at the transcript level. Evening Complex (EC) modulates Jasmonate-induced leaf senescence through transcriptionally repressing *MYC2*, an essential component of JA signaling pathway to trigger leaf senescence. TIC, which regulates circadian rhythm with unknown mechanism, can modulate protein abundance of MYC2 with an unclear post-translational mechanism. ELF3 also regulates dark induced leaf senescence (DIS) through ORE1-dependent pathway by transcriptionally repressing PIF4. Whether other circadian clock components, such as Pseudo Response Regulators, are engaged into the regulation of leaf senescence awaits to be further investigated.

Age-dependent leaf senescence is regarded as an autonomous signaling pathway to trigger the initiation of leaf senescence via reprogramming transcriptome. ORESARA 1 (ORE1/ANAC092), a NAC-domain transcription factor, has been characterized as a positive regulator for age induced senescence (Kim et al., 2009; Rauf et al., 2013). Elevated expression level of *ORE1* can trigger the onset of leaf senescence by inducing various SAGs expression. Importantly, ORE1 interacts with the G2-like transcription factors GLK1 and GLK2 and antagonizes their transcriptional activity, while GLKs are important for chloroplast development and maintenance (Rauf et al., 2013). At the transcriptional level, *ORE1* expression is regulated by age and multiple plant hormones, such as ABA and ethylene (Li et al., 2013). Recently, the tight circadian control of *ORE1* expression was unmasked to mediate circadian regulation on age dependent leaf senescence. By using high-resolution temporal profiling data, Song et al. (2018) showed that the expression level of *ORE1* is continuously increased with aging under long day condition, while the corresponding abundance of *CCA1* keeps reducing. In addition, they found that the expression level of *GLK2* keep decreasing during leaf senescence process (Song et al., 2018), suggesting their highly coordination might coincidence with the processing of leaf senescence. They further found that CCA1, the core circadian transcription repressor, can directly bind to the promoter of *ORE1* through its CBS element (CCA1-Binding site, AAMAATCT) and inhibits its transcription in the pre-senescence leaves (Song et al., 2018). Moreover, CCA1 is able to active *GLK2* expression through binding the CBS element within the *GLK2*’s promoter (Song et al., 2018). Hence, they proposed that the age-declined *CCA1* causes the gradual removal inhibition on *ORE1* transcription and the lower activation of *GLK2* to temporally promotes the onset of leaf senescence (**Figure 1**; Song et al., 2018).

Song et al. (2018) also showed the mutation of *ELF3* caused precocious leaf senescence, which might due to the aberrant expression of *ORE1*, because ELF3 forms transcription repressive complex together with ELF4 and LUX, to transcriptionally inhibit *PIF4* and *PIF5* expression. In turn, PIF4 can directly upregulate the transcription of *ORE1* (**Figure 1**; Sakuraba et al., 2017). However, the messenger RNA abundance of *PIF4* and *PIF5* increases with aging (Song et al., 2018), in contrast to that of *CCA1*. Previously, they have identified PIF3, PIF4, and PIF5 as mediators of leaf senescence as their overexpression promotes leaf senescence, while their mutation confers longer leaf longevity (Song et al., 2014). Thus, higher *PIF4* transcript level in *elf3* mutant can in turn cause the higher level of *ORE1* and bolster the onset of leaf senescence. Thus, it is reasonably to hypothesize that the higher level of *PIFs* in *elf3* mutant boosts up the leaf senescence through increasing *ORE1* expression, which need to be further verified experimentally. Taken together, the above lines of evidences strongly indicated the highly coordinated network underlying the circadian clock regulation on natural senescence, which remains to be fully investigated in the future.

## Circadian System Directs Plant Hormones Dependent Leaf Senescence

Jasmonoyl-isoleucine (JA-Ile, hereafter was abbreviated to JAs) has long been known as a key plant hormone to induce leaf senescence (He et al., 2002; Qi et al., 2015). In the absence of JA, JA ZIM-domain (JAZ) repressors interact with and inhibit JA downstream transcription factors, such as bHLH subgroup IIIe factors MYC2, MYC3, and MYC4, by recruiting TOPLESS co-transcriptional repressor family members, adapted by NINJA (Pauwels et al., 2010). On perception of JAs, its receptor, CORONATINE INSENSITIVE1 (COI1), which is a F-box domain containing protein, can interact with and degrade JAZ proteins, thus releasing transcriptional activators including MYC2, MYC3, and MYC4 to mediate JA response by activating downstream gene expression (Pauwels et al., 2010). Upon JA signaling activated, the IIIe bHLH factors MYC2, MYC3, and MYC4 redundantly activate JA-induced leaf senescence. On the other hand, the IIId bHLH factors including bHLH03, bHLH13, bHLH14, and bHLH17 acting redundantly to repress JA-Induced leaf senescence by antagonizing with MYCs, through competing with their binding *SAG29* promoter (Qi et al., 2015), suggesting the elaborative downstream regulation mechanism to mediate JA-induced leaf senescence. Nevertheless, MYC2, MYC3, and MYC4 play predominantly and positively regulatory roles in JA-induced leaf senescence.

A recent study from our laboratory details how *Arabidopsis* circadian system regulates JAs-induced senescence. We found that Evening Complex (EC), composed by ELF4-ELF3-LUX, locates at hub position to mediate circadian output regulation on JA-induced leaf senescence (Zhang et al., 2018). EC can directly bind to *MYC2* promoter and gate its JA-induced expression profile. Genetically, mutation of *MYCs* abolished the hypersensitivity of JA-induced leaf senescence in EC mutants (**Figure 1**; Zhang et al., 2018). This finding not only delineated a key underlying mechanism for circadian gated JA signaling in triggering leaf senescence, but also closed the knowledge gap of a long sought-after transcriptional regulation between the circadian core oscillator and JA signaling. Interestingly, JA accumulation in plants is also under the circadian control with peaking at the middle of subjective day. This phase pattern anticipates the peak of the insect feeding behavior (Goodspeed et al., 2012). But the JA content is lower in EC mutant, reflects the feedback regulation of elevated JA signaling, and further emphasizes the JA signal, rather than JA accumulation, accounts for precocious leaf senescence in EC mutant.

Circadian clock also regulates JA signaling and response at post-transcriptional level. TIME FOR COFFEE (TIC) has been identified as a nuclear regulator for proper circadian function from middle to late subjective night (Hall et al., 2003; Ding et al., 2007). TIC also acts as a negative regulator in JA signaling by repressing MYC2 protein accumulation at the post-translational level (Shin et al., 2012). Hence, the mutation of TIC caused higher MYC2 protein abundance and might cause early senescence phenotype, which awaits to be experimentally verified. Moreover, plant circadian clock driven time-of-day susceptibility to necrotrophic fungal pathogen, *Botrytis cinerea*, is mediated by JA signaling (Ingle et al., 2015), implicating that multiple converging points mediated circadian regualtion on JA responses. It is unclear yet whether this related to leaf senescence or not.

Salicylic acid (SA) is another well known plant hormone to positively regulate leaf senescence, and closely interplays with circadian clock. Plants with defective SA signaling, such as *npr1* (*non-expressor of pathogenesis-related gene 1*), display a delayed yellowing and reduced necrosis phenotype (Morris et al., 2000). Consistently, a few senescence enhanced genes, including *SENESCENCE ASSOCIATED GENE 12* (*SAG12*), display lower transcript level in *npr1* mutant, whereas SA treatment is capable of inducing a few senescence enhanced genes expression (Morris et al., 2000). Very recently, it was found that SA signal causes the reinforcement of circadian clock without circadian period change, instead of boosting circadian amplitudes of a few circadian core components via NPR1 (Zhou et al., 2015). Intriguingly, SA mediated plant immune response is influenced by circadian clock through the regulatory control of CIRCADIAN CLOCK ASSOCIATED 1 (CCA1) on expression of defense genes (Wang et al., 2011). However, whether SA mediated leaf senescence is governed by circadian clock or not still remains elusive, which will be of great interest to be fully investigated in the future.

## Circadian Clock Components Tune Environmental Stresses Dependent Leaf Senescence

Unfavorable environmental stresses can initiate the onset of leaf senescence. Among the stresses, light deprivation trigged leaf senescence was considered as highly relevant with circadian system, especially given the highly interconnection between light signaling and circadian clock. The Pfr form of Phytochrome B can inhibit dark induced leaf senescence (DIS), probably through destabilizing PIF4 and PIF5, as the Pr form of Phytochrome B could be switched off by far-red light. PHYTOCHROME INTERACTION FACTORS (PIFs), the negative regulator of light signaling regulator, regulate a variety of red light signaling mediated developmental responses. By physically interacting with Phy A and/or Phy B, PIFs will be degraded. Consistently, mutants of *PIF4* and *PIF5*, especially quadruple mutant of *PIFs*, displayed a significantly delayed DIS phenotype (Song et al., 2014; Sakuraba et al., 2017). ELF3 has known to be one of upstream regulators for *PIF4* and *PIF5* expression, in consistent with its mutant senesced much faster under dark induced condition. ELF3 functions as an adaptor to bridge ELF4 and LUX to form the ternary transcriptional complex to inhibit *PIF4* and *PIF5* expression in the early subjective night (Nusinow et al., 2011). Surprisingly, neither *ELF4* nor *LUX* mutants showed the faster DIS phenotype as mutant of *ELF3*, indicating the altered senescence phenotype in *elf3* mutant is not due to the transcription inhibition of *PIF4* and *PIF5*, instead it might be caused by post-transcriptional regulation of PIFs (**Figure 1**). Strikingly, none of any other circadian mutants tested by Sakuraba et al. (2017) displayed the altered DIS phenotype, including *CCA1*, *LHY*, *GI*. Intriguingly, ELF3 does not rely on the function of PhyB to inhibit DIS, but through transcriptionally repressing PIFs. Given both ELF4 and LUX did not involve DIS processing, whether ELF3 transcriptional repression activity for *PIFs* recruits tissue specific partners in light deprivation leaves remains an open question. Nonetheless, it is reasonable to hypothesize that the orchestration of PIFs abundance by light signaling at the post-translation level and circadian clock at the transcriptional level to direct PIFs mediated DIS. Mechanistically, increased both ethylene biosynthesis and signaling may also mediate the downstream events of PIFs to trigger DIS (Song et al., 2014; Sakuraba et al., 2017). In addition, ABA signaling was also involved into downstream of PIFs to mediate DIS, as ABSCISIC ACID INSENSITIVE 5 (ABI5) is direct target genes of PIF4 and PIF5 (Sakuraba et al., 2017; Zhang et al., 2018). Notably, PIFs and ABI5 regulate the expression of *ORE1*, the key senescence promoter, hence forming a coherent feed forward regulation mechanism (for review see Liebsch and Keech, 2016).

## Circadian Period Is Altered With Leaf Ages

Not only circadian clock regulates leaf senescence through a complex signaling network integrating the age-dependent, plant hormone mediated, and dark induced pathways to trigger the onset of leaf senescence, but also circadian clock itself was feedback regulated by the leaves senescing process. *SENESCENCE 4*(*SEN4*) is an age dependent marker gene whose expression is eventually decreasing in old leaves. While the younger leaves display the lower transcript level of *SEN4* in single plant, they also showed longer circadian period, indicating the close reverse association between leaf senescence and circadian period exists (Kim et al., 2016). Similarly, the measuring circadian period of the detached third and fourth leaves from plants at different ages with dramatically distinct *SEN4* expression level further confirmed that the younger leaves have a relatively longer period, while the old leaves with a shortened period (Kim et al., 2016). Intriguingly, the gradual decrease of circadian period correlates to the day length as the more pronounced circadian period change was found in long-day grown plants. Among the tested circadian mutant, *toc1* mutants did not display shorter circadian period with aging, implicating TOC1 is an essential player to link leaf aging with circadian period change (**Figure 1**; Kim et al., 2016). However, the underlying mechanism how TOC1 mediates the link is unclear yet. Moreover, whether abiotic stresses or hormones induced senescent leaves will also alter circadian period needs to be further explored.

## Perspectives and Remaining Questions

In conclusion, the intricate complex cellular network, but not linear signaling pathways, may govern the regulation of circadian clock on the initiation and processing of leaf senescence. As an example, besides promoting the DIS, PIFs also promote age-dependent leaf senescence which may act through activating ethylene and JA signaling pathway (Sakuraba et al., 2014; Song et al., 2014). This also could be true for explaining EC regulation on leaf senescence. As JA content does not increase with aging (Mao et al., 2017) and JA response declining with age, the early senescence phenotype in EC mutant under nature condition cannot simply caused by elevated expression level of *MYC2*. Actually, *ABI5*, *WRKY22* and a few ethylene response regulators were also appreciably up-regulated in *lux-6* mutant (Zhang et al., 2018).

On the other hand, since ABA-responsive genes are also circadian regulated, either as direct targets of the central clock or as a response of the rhythmic ABA signaling network (Seung et al., 2012; Liu et al., 2013; also for review see Sanchez and Kay, 2016). Whether ABA signaling is under the circadian control to timing leaf senescence awaits to be further investigated.

Moreover, Reactive oxygen species (ROS) homeostasis is essential for triggering leaf senescence, while the cellular redox state and circadian clock daily influence each other, partially via *CCA1* (Lai et al., 2012). It is thus conceivable that circadian direct regulation on ROS homeostasis might also contribute the onset of leaf senescence bypass the known signaling pathways. Nevertheless, circadian clock acts as a master shepherd for timing and phasing the onset of leaf senescence, and the underlying regulatory network remains to be further discovered.

Another challenge will be to understand the cause and effect relationship between senescence and circadian clock. It remains unclear whether the age dependent change of circadian rhythm will induce downstream developmental program which further trigger the leaf senescence due to mis-synchronization or aged leaves has lower energy status and higher ROS accumulation which will cause the change of circadian period. It also cannot exclude the possibility that the leaves senescing process and circadian rhythm work interactively with aging. Recently, It was found that disruption peripheral circadian clock in mammals can reduce life span and accelerate aging (for review see Paschos and FitzGerald, 2017). Thus, circadian clock, the central pacemaker, tightly interconnected with regulatory network of leaf senescence by directing plant hormone response and ROS homeostasis, etc., to ensure the plant proper fitness and yield. The complex underlying regulatory network awaits to be further fully elucidated.

## Author Contributions

YW, YZ, and LW wrote the manuscript and contributed to the discussion and approved the final manuscript.

## Conflict of Interest Statement

The authors declare that the research was conducted in the absence of any commercial or financial relationships that could be construed as a potential conflict of interest.
